# Necrostatin-1 promotes ectopic periodontal tissue like structure regeneration in LPS-treated PDLSCs

**DOI:** 10.1371/journal.pone.0207760

**Published:** 2018-11-21

**Authors:** Bingbing Yan, Hongmei Zhang, Taiqiang Dai, Yongchun Gu, Xinyu Qiu, Cheng Hu, Yan Liu, Kewen Wei, Dehua Li

**Affiliations:** 1 State Key Laboratory of Military Stomatology & National Clinical Research Center for Oral Diseases & Shaanxi Engineering Research Center for Dental Materials and Advanced Manufacture, Department of Oral Implants, School of Stomatology, The Fourth Military Medical University, Xi’an, Shaanxi, China; 2 Department of Burns and Plastic Surgery, Tangdu Hospital, The Fourth Military Medical University, Xi'an, Shaanxi, China; 3 State Key Laboratory of Military Stomatology & National Clinical Research Center for Oral Diseases & Shaanxi Clinical Research Center for Oral Diseases, Department of Oral and Maxillofacial Surgery, School of Stomatology, The Fourth Military Medical University, Xi’an, Shaanxi, China; 4 Department of Dentistry, First people's Hospital of Wujiang Dist, Nantong University, Suzhou, China; 5 Research and Development Center of Tissue Engineering, School of Stomatology, The Fourth Military Medical University, Xi’an, Shaanxi, China; 6 Department of Orthodontics, Stomatology Hospital of Xi'an Jiaotong University College of Medicine, Xi'an, Shaanxi, China; 7 State Key Laboratory of Military Stomatology & National Clinical Research Center for Oral Diseases & Shaanxi Key Laboratory of Stomatology, Department of Prosthodontics, School of Stomatology, The Fourth Military Medical University, Xi’an, China; Kanagawa Dental University, JAPAN

## Abstract

Necroptosis is a programmed necrosis, regulated by receptor interacting protein kinase 1(RIP1) and receptor interacting protein kinase 3(RIP3), and could be inhibited by necrostatin-1(Nec-1) specifically. This study aims to evaluate the effect of Nec-1 on LPS-treated periodontal ligament stem cells (PDLSCs). In the research, three groups were established: normal cultured PDLSCs, Porphyromonas gingivalis (Pg)-LPS stimulated PDLSCs and Pg-LPS+Nec-1 treated PDLSCs. The expression of RIP1 and RIP3 and osteogenic differentiation of PDLSCs in three groups were analyzed. Then, we constructed cell aggregates (CA) using PDLSCs, then PDLSCs-CA were combined with Bio-Oss in three groups were transplanted subcutaneously in nude mice to assess their potentials of periodontal tissue regeneration. The results showed that RIP1 and RIP3 were fully expressed in Pg-LPS stimulated PDLSCs and the level increased significantly. Nec-1 inhibited RIP1-RIP3 interaction, and further inhibited necroptosis of PDLSCs in inflammatory state. Moreover, Nec-1 pretreatment ameliorates the osteogenic differentiation of LPS-treated PDLSCs and can effectively promote the cementum like structure ectopic regenerative ability of PDLSCs in nude mice. These findings show RIP1/RIP3-mediated necroptosis is an important mechanism of cell death in PDLSCs. Nec-1 has a protective effect in reducing cell death and promotes ectopic periodontal tissue like structure regeneration by inhibiting necroptosis. Nec-1 is a hopeful therapeutic agent which protects cells from necroptosis and ameliorates functional outcome.

## Introduction

Periodontitis is one of the most common oral diseases in adults, and also the leading cause of adult tooth loss [1]. Incidence rate of periodontitis has been found as high as 50%, and moreover, it can increase the risk of heart disease, diabetes, arthritis, as well as the pregnancy complications[2].Chronic periodontitis is a periodontal tissue destructive disease which is associated with immune reaction. Its pathogenesis is due to the bacteria or their products that gather on the surface of tooth roots or periodontal tissue. It invades periodontal tissue, leading to chronic inflammation, absorption of alveolar bone and finally resulting in tooth loss [3]. The ultimate objective of periodontitis treatment is to realize the regeneration of periodontal tissue in inflammatory environment and restore the structure and function of periodontal tissue [4, 5].

PDLSCs play an important role in periodontal regeneration. They can differentiate into periodontal membrane cells and cementum cells and form the tissue of cementum /periodontal structure either in vitro, or in vivo [6, 7]. Therefore, PDLSCs are regarded as ideal candidate cell sources for periodontal tissue regeneration. However, previous studies demonstrated that PDLSCs differentiation and periodontal tissue regeneration ability declined in the inflammatory microenvironment, and the underlying molecular mechanism was still to be elucidated [8, 9]. Therefore, how to regulate inflammatory response and promote periodontal regeneration is an urgent problem to be solved and is of great important significance [10]. Pg-LPS is the main pathogen of chronic periodontitis [11]. Therefore, in this study we simulated periodontal inflammatory conditions by treatment of PDLSCs with Pg-LPS [12].

Necroptosis is a caspase-independent cell death type which is activated when caspase is inhibited or not activated [13]. Recently, several studies have shown necroptosis is regulated by RIP1 and its interaction with RIP3 after death signal stimulated [14]. Nec-1, which is necroptosis' specific small molecule inhibitor, could specially inhibit RIP1 kinase activity and the interaction of RIP1-RIP3 [15, 16].Necroptosis has been shown to participate in various disease models, including periodontal disease [17]. However, it is still required to be elucidated whether RIP1/RIP3-mediated necroptosis occurs in LPS-treated PDLSCs and whether Nec-1 could inhibit necroptosis of PDLSCs and promote periodontal tissue like structure ectopic regeneration.

In this study, we found RIP1/RIP3-mediated necroptosis contributed necrosis of LPS-treated PDLSCs. Furthermore, we proved that RIP1 kinase inhibitor Nec-1 ameliorated PDLSCs' ectopic periodontal tissue like structure regeneration ability through inhibiting RIP1/RIP3 pathway.

## Materials and methods

### Reagents and antibodies

Pg-LPS was bought from Sigma (St. Louis, MO, USA). Rabbit RIP1, MLKL and beta-actin antibody was from Proteintech (USA), and mouse RIP3 antibody was from Santa (USA). Real-time PCR primer COL-1, OCN, RUNX-2, BSP, TNF-α, IL-1β, IL-18 and GAPDH were from Genecopoeia (USA), SYBR mixture was from Takara (Japan), RT Master Mix was from Takara (Japan). Inhibitor of Nec-1 (ab141053) was from Abcam (USA). Annexin V-FITC/PI: Cell apoptosis detection kit was from Bestbio Company (China). Nude mice were bought from Animal Center of the Fourth Military Medical University Animal Center.

### Animals

This study was performed with the approval of the Institutional Animal Care and Use Committee of the Fourth Military Medical University (Protocol Number: 20170605), China. Nine male BALB/c strain nude mice(8-week-old, weight 20-25g), specific-pathogen-free, were obtained from Animal center of the Fourth Military Medical University and they were kept by professional staff. The stream of people, material, animal and air were rescuable. All of the mice received humane care following the Guide for the Care and Use of Laboratory Animals of the National Institutes of Health. Mice were housed in a temperature and humidity controlled room with an artificial 12-h light/dark cycle and provided free access to food and water. The mice were acclimated for one week prior to the study.

### Culture of PDLSCs

All experiments were performed with the approval of the Ethics Committee of Stomatology College of Fourth Military Medical University (FMMU), and each participants sign a written content (Approval Number: 2016051). Healthy teeth of 18–25 year old orthodontic patients were extracted (n = 3) at the Department of Maxillofacial Surgery, Qindu Stomatology Hospital, Xian, China. All the cells used in this study were at passages 2–4. PDLSCs were isolated and cultured as described preciously [6, 7]. Briefly, periodontal ligament (PDL) was carefully scraped and digested in 3mg/ml collagenase type I (Worthington Biochem) for 15 min at 37°C with 5% CO₂, Single-cell suspensions were obtained by passing the cells through a 70μm strainer (BD Labware) and then was cultured at 37°C in 5% CO₂ using 10% fetal bovine serum (FBS) α-MEM (Invitrogen) supplemented with penicillin/streptomycin (Invitrogen). Cell culture medium was changed every three days. 1×10^6^ PDLSCs were harvested. Then the single-cell suspension was re-suspended and stained with mesenchymal stem cell surface markers, including Stro-1 (PE), CD146 (PE), CD34 (PE) and CD45 (APC) (e Bioscience) at 4°C and identified with flow cytomerty (Beckman Coulter, Fullerton, CA, USA). The experiment was repeated 3 times.

### In vitro osteogenic assay

Cells were cultured in osteogenic medium. Basic medium enclosed with 50 mg/mL L-ascorbic-2-phosphate (MP Biomedicals, LLC, Santa Ana, CA, USA), 0.1mM dexamethasone, and 5mM β-glycerophosphate (Sigma Aldrich). 10^6^ PDLSCs in 10cm plates for 7 day's induction, BCIP/NBT ALP color development KIT (Beyotime, Shanghai, China) was taken to determine the capacity of osteogenesis differentiation of PDLSCs abiding by the manufacturer’s instruction, and the quantification assay was carried out by an ALP activity detection Kit (Jiancheng Bioengineering, Nanjing, China), and Plus 5.0 software (Media Cybernetics, USA). After 28 day's induction, the cells were fixed with 4% paraformaldehyde for 20 min, and stained with 2% Alizarin Red (PH 4.2) (Kermel, Tianjin, China). Dissolving the mineralized nodules with hexadecylpyridinium chloride and isopropanol, and quantitative absorbance was measured at 560nm for statistical analysis.

### Cell treatment

PDLSCs were divided into three groups:(a)control group: PDLSCs were cultured in normal α-MEM medium, (b) Pg-LPS group: PDLSCs were cultured in medium containing Pg-LPS (10 μg/mL), and (c) necroptosis inhibited group (Pg-LPS+Nec-1): PDLSCs were cultured in medium containing Pg-LPS (10 μg/mL) and Nec-1(30 μM).

### Transmission electron microscope

The cells were fixed with glutaraldehyde and then were dehydrated, permeated, buried, hyperbo and lead uranium. The specimen was then observed under the transmission electron microscope (JEOL-1230, LSCM, Hitachi, Japan).

### Immunoprecipitation

The sample cells were washed 3 times with PBS and lysed with RIPA buffer. After centrifugation at 12 000 rpm for 15 min at 4°C, the supernatant were collected Protein concentrations were quantified by BCA kit (Thermo Scientific). RIP1 was immunoprecipated using Pierce Co-IP Kit (Thermo Scientific, 26149) following manufacturer's instructions. Brifely, protein in the lysate were crosslinked with aminolinkagarose resin for 2 hours. Binding of anti-RIP1 antibody or IgG (Santa Cruz, USA) were performed at 4°C overnight before wash and elution. The precipated protein samples were analyzed by Western blotting.

Samples were boiled in SDS buffer, run on SDS-PAGE gels and transferred to nitrocellulose membranes. After blocking with 5% non-fat dry milk in TBST for 1 hour at room temperature, membranes were incubated overnight at 4°C with anti-RIP1 (Proteintech, USA), anti-RIP3 (Santa, USA) antibody or β-actin (abcam, USA) with a concentration of 1:1000 in TBST. A concentration of 1:10,000 horseradish peroxidase-conjugated secondary antibody was used to incubate the membranes for 1 hour at room temperature. Membranes were incubated with TMB chromogenic solution to detect the target proteins.

### Quantitative analysis of apoptosis

Apoptosis of PDLSCs was detected by the Annexin-V-PI apoptosis staining kit (Bestbio, China) and then analyzed by FACS Calibur flow cytometer. Four cell populations can be identified: the viable population in the lower-left quadrant (Annexin-/PI-), the early apoptotic cells in the lower-right quadrant (Annexin+/PI-), the late apoptotic or necrotic population in the upper-left quadrant (Annexin+/PI+), and the necrotic cells population in the upper-left quadrant (Annexin-/PI+).

### Real-time quantitative polymerase chain reaction (PCR)

After 7 days’ osteogenesis induction, the expression of COL-1, OCN, RUNX-2 and BSP in PDLSCs were detected by real-time PCR. Total cellular RNA was extracted using Trizol Reagent (Omega Bio-tek, USA). Obtaining cDNA used a DNA synthesis kit (Takara, Bio, Otsu, Japan). Performing quantitative real-time PCR used SYBR qRT-PCR kit (Takara, Japan) following the manufacturer's instructions. Primers were synthesized as follows:

GAPDH-F: GCACCGTCAAGGCTGAGAAC, GAPDH-R: TGGTGAAGACGCCAGTGGA; COL-1-F: AAGGTGTTGTGCGATGACG, COL-1-R: CAGACGGGACAGCACTCG;

RUNX-2-F:TGACCATAACCGTCTTCACAA,RUNX-2-R:GGTTCCCGAGGTCCATCTA;OCN-F:GAGGGCAGCGAGGTAGTG,OCN-R:CCTGAAAGCCGATGTGGT,BSP-F:GAGGGCAGCGAGGTAGTG,BSP-R:CCTGAAAGCCGATGTGGT.

Total RNA concentrations from each sample were normalized by quantity of GAPDH mRNA, and the expression levels of target genes were evaluated by ratio of the number of target mRNA to GAPDH mRNA. The company of PCR machine is C1000 Thermal Cycler (USA), and it takes 40 cycles, 95°C for 15 seconds, and 60°C for one minute.

### Immunofluorescence

Immunocytochemical analysis of RIP1 and RIP3 were performed using a standard protocol. The cells were fixed with 4% paraformaldehyde and permeabilized with 0.2% Triton X-100 (Sigma) for 15 min. Then they were blocked with goat serum and incubated with primary antibodies against RIP1 (Proteintech, USA) (1:100) and RIP3 (Santa, USA) (1:200) overnight at 4°C. After that, cells were further incubated with Alexis Fluor 488-conjugated anti-rabbit IgG and anti-mouse IgG secondary antibody (Zhuangzhibio, China) (1:100), respectively. Nuclei were stained with DAPI (Beyotime, China). Immunofluorescent images were captured using a fluorescence microscope (Olympus IX71, Japan).

### In vivo transplantation

Before transplantation, 4×10^5^ PDLSCs (passage4) were cultured with a membrane-inducing medium (α-MEM nutrient solution which contains 10% FBS and 50 μg/mL L-ascorbic acid phosphate (Wako, Japan) in 6-well plates for 7 days. Then, Pg-LPS or Nec -1 were added to each group at the corresponding concentration for 24 hours. Then the PDLSCs were induced to form complete cell aggregate that could be detached at the edge of the dishes and showed ivory and membrane-like morphologies. The cell aggregate (3 sheets) mixed with 40mg Bio-Oss (Geistlich, Switerland) was transplanted subcutaneously into the dorsal surface of 9 nude mice (3 animals per testing group) as previously described[18]. The maximum size of PDLSC implants in vivo is 6 mm *4 mm. No mice died accidently or for other factors in the process of experiment, and finally, all the mice entered the stage of result analysis. Mice were anesthetized by intraperitoneal injection of 40 mg/kg 1% sodium pentobarbital, and we made all efforts to minimize suffering. After 8 weeks, the mice were humanely euthanized under deep anesthesia with a cocktail of ketamine and xylazine (100/10 mg/kg) according to approved IACUC guidelines. The transplants were harvested for histological analysis.

### Haematoxylin-Eosin staining and Masson trichrome staining

The transplants, fixed with 4% formalin, were decalcified with 10% EDTA (PH = 8.0), and then embedded in paraffin. Sections were deparaffinised and stained with Haematoxylin-Eosin (HE) and Masson trichrome (Sigma, HT200-1KT, USA). New cementum was defined as the mineralized tissue formed by collagen bundles, and the structure is similar to the Sharbey’s fibers buried in the cement. The percentage of new cementum was calculated by dividing the length of the whole area by the length of proportion with new cementum.

### Statistical analysis

Data were showed as means ± SEM. We analyzed the statistical results using GraphPad Prism 6 software. One-way or two-way analysis of variance followed by the Bonferroni's post hoc test was used to compare groups. Statistical significance was set at p< 0.05.

## Results

### 1 PDLSCs culture & identification

**Fig 1:**
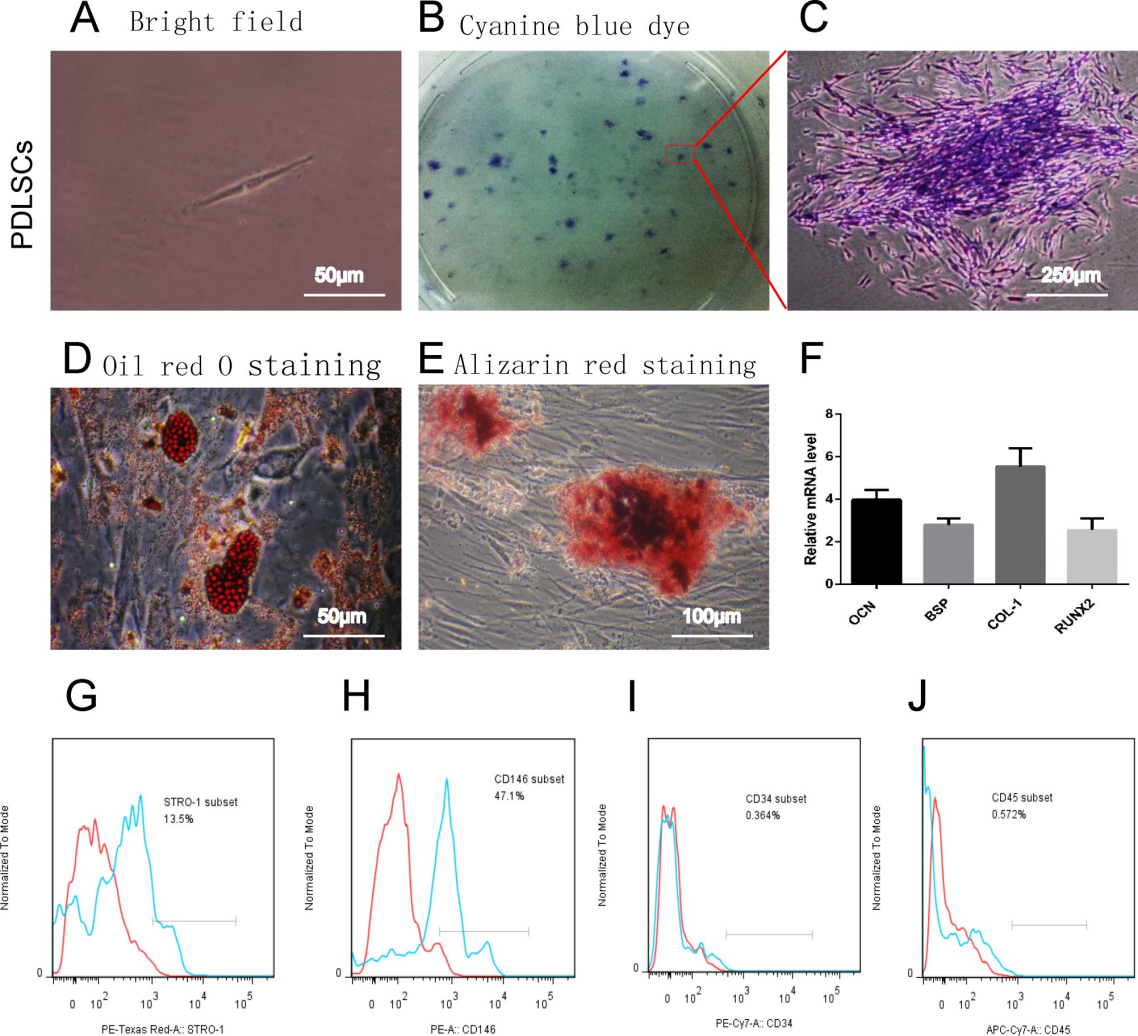
PDLSCs culture & identification. (A)single PDLSCs, (B) PDLSCs polyclonal formation, (C) a clone of PDLSCs stained by the cyanine blue, (D) PDLSCs lipid droplet formed after lipid induction, oil red O staining positive, (E) PDLSCs after osteo-induction, mineralized nodules were formed, and alizarin red staining was positive. (F) RT-PCR after mineralized induction, PDLSCs expressed osteogenic genes(COL1, OCN, RUNX-2, BSP); (G and H) Flow cytometry detection of the mesenchymal stem cell marker shows PDLSCs are positive for STRO-1 and CD146, and (I and J) negative for CD34 and CD45.

1000 PDLSCs(Fig 1A) were inoculated in each culture dish(10cm) for 10 days and (97.4±2.8) coloniese were formed (Fig 1B and 1C).Clonal cultured PDLSCs have multi-directional differentiation ability: osteogenic and adipogenic differentiation (Fig 1D and 1E), and they expressed surface markers of the mesenchymal stem cell marker: STRO-1 and CD146 (Fig 1G and 1H), but negative for the hematopoietic markers of CD34 and CD45 (Fig 1I and 1J); After mineralization induction, RT-PCR detected the expression of osteogenic mRNA: COL-1, OCN, RUNX-2, BSP (Fig 1F). All of these show that PDLSCs are consistent with the characteristics of mesenchymal source.

### 2 Necroptosis is involved in cell death of LPS-treated PDLSCs

**Fig 2:**
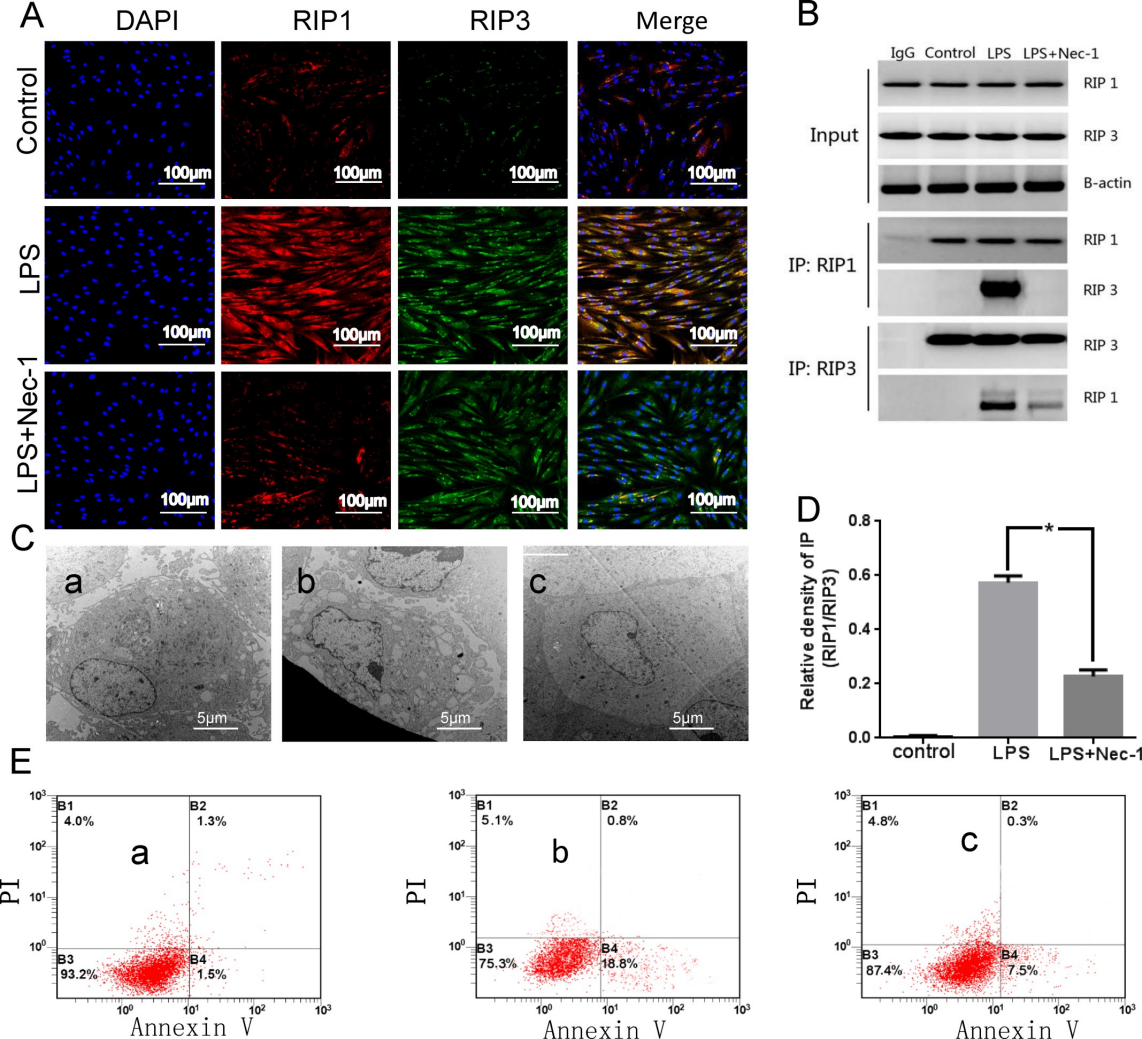
Necroptosis exists in PDLSCs. (A) immunofluorscence staining of RIP1 and RIP3. (B) CO-IP, a representative immunoprecipitation (IP) and western blot (WB) results for RIP1 and RIP3 interaction. IgG as a negative control. RIP1 and β-actin input as loading control. (C) TEM, a representative normal cell (the cell had a long fusiform, cell membrane structure was complete, the nucleus was round, nucleolus was obvious and center), b representative necroptosis cell (cell membrane ruptured, organelle disappeared, chromatin consolidated and agglutinated, presenting necrotic ultrastructural characteristics), c representative necrosis cell after Nec-1 inhibited (the cell membrane was intact, and the chromatin structure returned to normal). (D) Quantification of CO-IP showing RIP1 and RIP3 interaction is significantly decreased after Nec-1 pretreatment. *p<0.05 versus LPS. (E) Annexin V, PI/FITC FCM. Quantitative analysis of apoptosis. a control group, b Pg-LPS group, c Pg-LPS+Nec-1 group.

Immunofluorscence data showed an increased expression of RIP1 and RIP3 in Pg-LPS group and an eminent reduction of RIP1 in the Pg-LPS+Nec-1 group. In agreement with this finding, we found that Nec-1 had impaired necroptotic signaling in vitro, which indicated RIP1 and RIP3 could be expressed in the same part of the cells (Fig 2A).

Consistent with immunofluorscence findings, we detected RIP1/RIP3 complex with Co-immunoprecipitation technique. To test whether Nec-1 pretreatment had any effect on RIP1-RIP3 interaction in PDLSCs, we investigated the recruitment of RIP3 to RIP1 by immunoprecipitation. Our result showed that the interaction between RIP1 and RIP3 was greatly enforced in the Pg-LPS group. However, Nec-1 pretreatment could significantly reduce RIP3 recruitment to RIP1 in Pg-LPS-induced PDLSCs' microenvironment (Fig 2B and 2D).

TEM investigation detected necroptosis in the Pg-LPS group (cell membrane ruptured, organelle disappeared, chromatin consolidated and agglutinated, presenting necrotic ultrastructural characteristics), whereas in the control group, the cell morphology is normal (the cell had a long fusiform, cell membrane structure was complete, the nucleus was round, nucleolus was obvious and center). The third group showed Nec-1 treatment could inhibit the degree of necroptosis, and the cell membrane was intact, and the chromatin structure returned to normal (Fig 2C).

Flow cytometry analysis (PI/Annexin V staining) showed: under Pg-LPS stimulation, cell necrosis increased, while cell necrosis relieved after treatment with necroptosis inhibitor. The results showed that necroptosis was found existing in LPS-treated PDLSCs (Fig 2E).

### 3 Nec-1 ameliorates the osteogenic differentiation of LPS-treated PDLSCs

**Fig 3:**
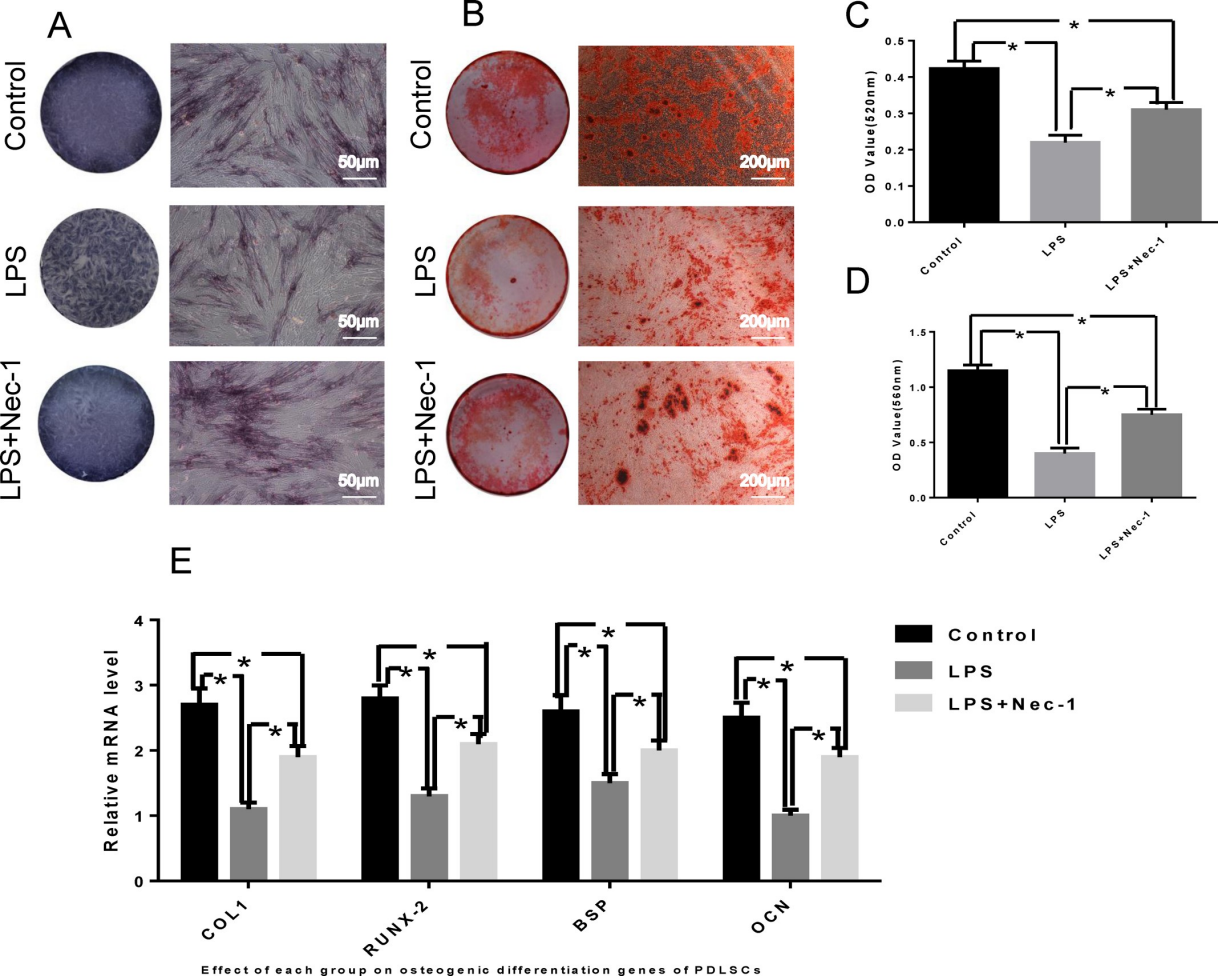
Osteogenic differentiation. (A) Representative images of alkaline phosphatase (ALP) staining in PDLSCs treated by osteogenic induction medium for 7 days. (B) Cultured PDLSCs form calcified nodules that stained positively for Alizarin Red S staining after 4 weeks of osteogenic induction. (C) Quantitative analysis of ALP activity in PDLSCs. (D) Quantitative comparison of mineralized nodule formation. (E) The relative mRNA levels of osteogenic related gene. *: p < 0.05.

After being cultured in osteo-inductive medium, PDLSCs showed increased ALP expression level (Fig 3A), and mineralized extracellular matrices were observed in PDLSCs, as demonstrated by Alizarin Red Staining (Fig 3B). Quantitative analysis showed that PDLSCs had decreased osteogenic potential under chronic inflammatory conditions (Fig 3C and 3D), but this trend can be reversed by treatment of Nec-1.

LPS-treated PDLSCs were cultured in vitro, Nec-1 signal inhibition, and the osteo-induction solution were added to detect the osteogenic differentiation of PDLSCs, and the expression level of the related osteogenic genes in osteogenic differentiation was detected by real-time PCR. The results showed that PDLSCs osteogenic capacity declined after Pg-LPS stimulation, whereas Nec-1 could ameliorate the decline tendency. As shown in Fig 3E.

### 4 Nec-1 can effectively promote the ectopic regeneration of periodontal tissue-like structure of LPS-treated PDLSCs in nude mice

**Fig 4:**
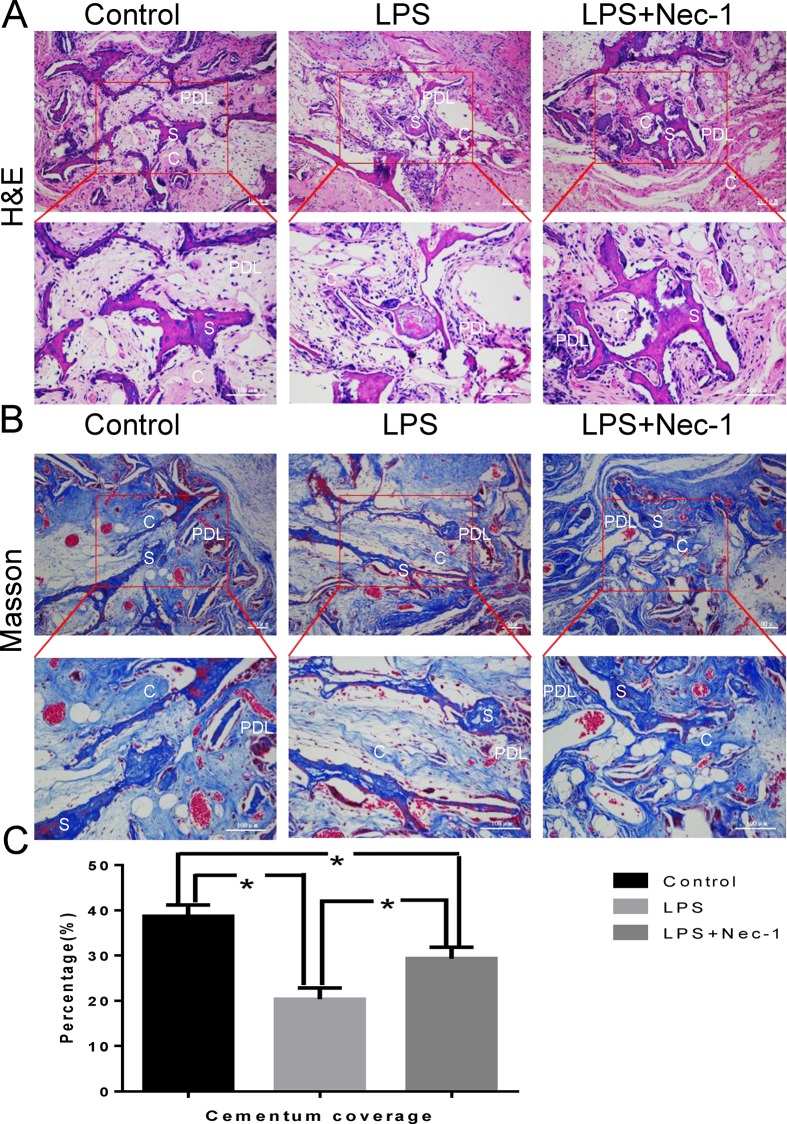
Histomorphometric analysis of the newly formed cementum-like mineralized deposits tissues. Representative hematoxylin and eosin (H&E) Staining (A) and Masson Staining (B) indicated the formation of cementum-like tissue and periodontal ligament fibers at 8 weeks after surgery. (C) The corresponding quantitative analysis of histomorphometry observation. C: cementum-like tissue; S: scaffold; PDL: periodontal ligament. *: p<0.05.

HE and Masson staining results showed that there was osteoid tissue formation in all three groups. The bone material in the sample is wrapped around by the outer cytomembrane, and the stem cells migrate into the hollow part of the scaffold material through the cells, forming the skeletal structure. Under the microscope, there is a decrease in cementum-like tissue mass, however, the amount of the cementum-like tissue in Nec-1 inhibition group was significantly higher than that of the Pg-LPS group (Fig 4). These findings demonstrated that the LPS-treated environment can compromise the ability of the PDLSCs to form heterotopic cementum-like tissue, whereas the Nec-1 could rescue the cementum-like tissue formation ability of the PDSCs effectively.

## Discussion

Necroptosis is recently discovered programmed necrosis, and it is activated by TNF-α and/or Fas [19, 20]. A growing number of researches indicate necroptosis, distinct from apoptosis and necrosis, contributes to a range of human diseases as a new mechanism of cell death. Necroptosis has been found playing important roles in different pathophysiological conditions, including viral infection, ischaemic heart disease and brain death [15, 16]. In the case of necroptosis, there is no an end index like DNA fragment specificity which is in the process of apoptosis [21]. In general, the morphological changes (including TEM and PI staining positive) combined with biochemical method are often regarded as the basic criterion for necroptosis (which can be blocked by Nec-1 or knocking out of RIP1, RIP3), and RIP1-RIP3 signal compounds "necrosome” formation, RIP1 and RIP3 have mutual phosphorylation as complementary judgment index. As a result, TEM can demonstrate that necroptosis happened in PDLSCs after Pg-LPS stimulation, and the state of cell necrosis was significantly improved after treatment with Nec-1. FCM analysis of apoptosis (Annexin V, PI/FITC) showed that necroptosis occurred in PDLSCs in the inflammatory microenvironment, as Nec-1 is the specific inhibitor for necroptosis. Co-IP results showed RIP1-RIP3 had mutual phosphorylation and formed signal compounds "necrosome”. Immunofluorescence screening is an important supplement for the diagnosis of necroptosis, and it can reveal co-localization of RIP1 and RIP3 in the same cell, which provides the possibility for further integration.

PDLSCs can expand in vivo after they were transplanted into nude mice, and they can form tissue which are similar to periodontal membranes. The texture is similar to structure of cementum or periodontal ligament, and it's especially important that the fiber can be embedded into the structure of cementum to form sharpey-like structure [18]. The formation of this structure is considered to be necessary for functional periodontal tissue regeneration. Our results proved that pretreatment with Nec-1 obviously promoted the ectopic regeneration of periodontal tissue like structure (Fig 4). By suppressing RIP1 kinase activity and RIP1-RIP3 interaction in vitro studies, we obtained enough evidences to show that Nec-1 could inhibits necroptosis. We found RIP1 recruited RIP3 and formed RIP1-RIP3 complex in inflammatory state of PDLSCs (Fig 2B).Nec-1 treatment distinctly suspended the pro-necrotic RIP1-RIP3 complex formation (Fig 2A). We found the same results from the in vitro osteogenic differentiation studies (Fig 3). These results collectively indicated that RIP1 and RIP3 were critical regulators for necroptosis. In Pg-LPS-induced state of PDLSCs, inhibition of RIP1-RIP3 interaction might be the main mechanism of Nec-1 medicated periodontal protection.

This study reveals the mechanisms of RIP1/RIP3-mediated necroptosis in Pg-LPS-induced PDLSCs. However, further work needs to identify whether other necroptosis signaling pathways participate in LPS-treated PDLSCs [22]. In general, we demonstrated necroptosis may be a crucial pathogenic mechanism of cell death in LPS-treated PDLSCs, and both in vitro and in vivo experiments have confirmed that Nec-1 reduce cell death and RIP1-RIP3 interaction, and further inhibit the inflammatory response in PDLSCs (Fig 2C and 2E). Moreover, Nec-1 can block necroptosis of PDLSCs, thereby alleviating the decline of osteogenic differentiation capacity (Fig 3). Collectively, all those results demonstrated that Nec-1 attenuated LPS-treated PDLSCs’ ectopic periodontal tissue like structure destruction by inhibiting RIP1/RIP3-mediated necroptosis.

In summary, we demonstrate that Nec-1, which is RIP1's special inhibitor, can effectively block PDLSCs’ necroptosis through suppressing RIP1-RIP3 interaction. This is the first systematic study of necroptosis as a new way of cell death pattern in PDLSCs.

## Supporting information

S1 Checklist The ARRIVE guidelines checklist. (DOCX)
