# Supplementary material for: Necrostatin-1 promotes ectopic periodontal tissue like structure regeneration in LPS-treated PDLSCs
Source: PLoS One. 2018 Nov 21;13(11):e0207760. doi: 10.1371/journal.pone.0207760 (PMC6248998; doi:10.1371/journal.pone.0207760)

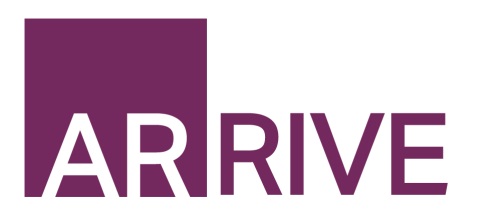


The ARRIVE Guidelines Checklist

Animal Research: Reporting In Vivo Experiments

Bingbing Yan ^1,☯^, Hongmei Zhang^2,☯^, Taiqiang Dai^3^, Yongchun Gu^4^, Xinyu Qiu^5^, Cheng Hu^6^, Yan Liu^7^, Kewen Wei^2,^*, Dehua Li^1,^*

*^1^ State Key Laboratory of Military Stomatology &National Clinical Research Center for Oral Diseases&Shaanxi Engineering Research Center for Dental Materials and Advanced Manufacture,* *Department of Oral Implants，School of Stomatology, The Fourth Military Medical University, 145 West Changle Road, Xi'an,* *Shaanxi710032, China*

*^2^* *Department of Burns and Plastic Surgery, Tangdu Hospital, The Fourth Military Medical University, 145 West Changle Road, Xi'an,Shaanxi710038, China*

*^3^ State Key Laboratory of Military Stomatology &National Clinical Research Center for Oral Diseases& Shaanxi Clinical Research Center for Oral Diseases, Department of Oral and Maxillofacial Surgery，School of Stomatology, The Fourth Military Medical University, 145 West Changle Road, Xi'an ,Shaanxi710032, China*

*^4^ Department of Dentistry,* *First people's hospital of Wujiang Dist, Nantong University, Suzhou215200, China*

*^5^ Research and Development Center of Tissue Engineering, School of Stomatology, The Fourth Military Medical University, 145 West Changle Road, Xi'an, Shaanxi710032, China*

*^6^ Department of Orthodontics, Stomatology Hospital of Xi'an Jiaotong University College of Medicine, Xi'an, Shaanxi710032, China.*

*^7^ State Key Laboratory of Military Stomatology & National Clinical Research Center for Oral Diseases & Shaanxi Key Laboratory of Stomatology, Department of Prosthodontics, School of Stomatology, Fourth Military Medical University, Xi’an, China.*

|  | | ITEM | RECOMMENDATION | | Section/ Paragraph |
| --- | --- | --- | --- | --- | --- |
| 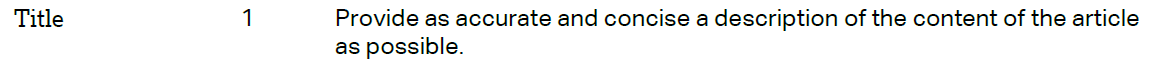 | | | Title | |  |
| 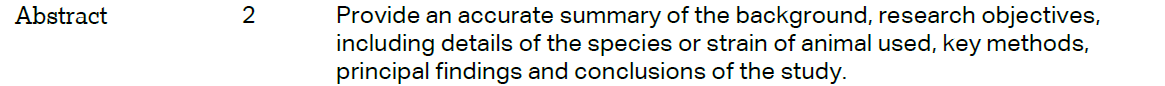 | | | Abstract | |  |
| INTRODUCTION | | |  | |  |
| 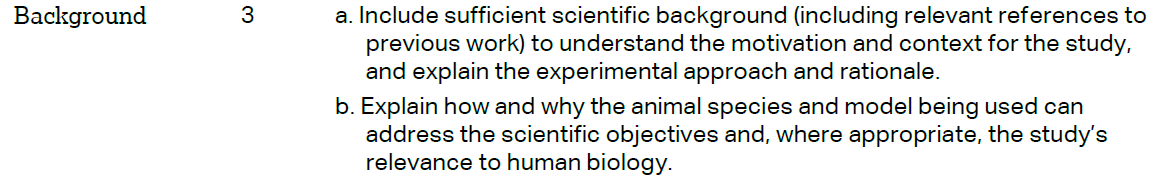 | | | Paragraphs 1-3 | |  |
| 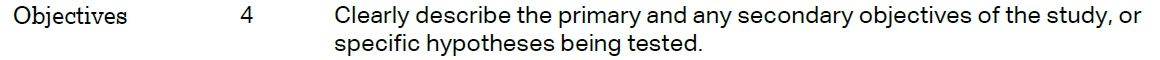 | | | Paragraph 4 | |  |
| METHODS | | |  | |  |
| 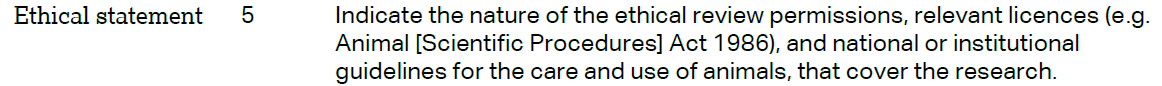 | | | Paragraph 2 | |  |
| 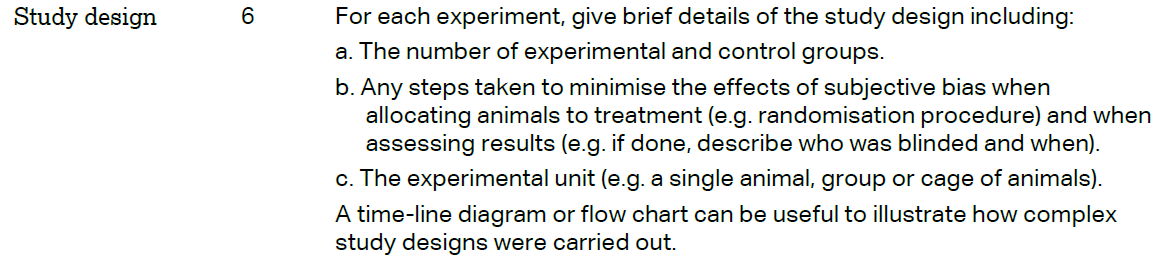 | | | Paragraph 5  Paragraph 12  Paragraph 12 | |  |
| 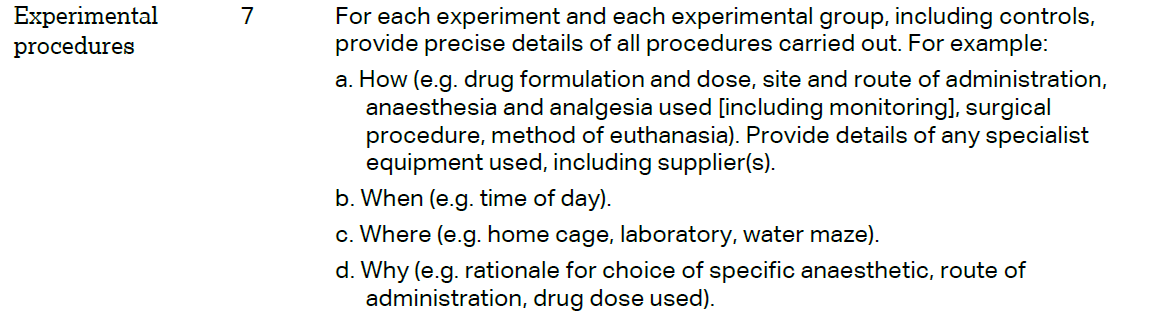 | | | Paragraph 12  Paragraph 2  Paragraph 12 | |  |
| 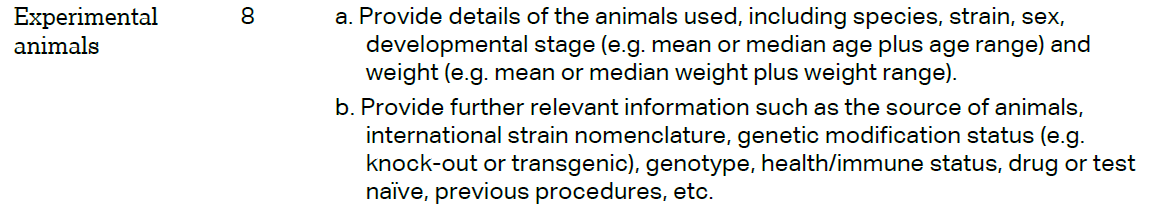 | | | Paragraph 2  Paragraph 2 | |  |

| 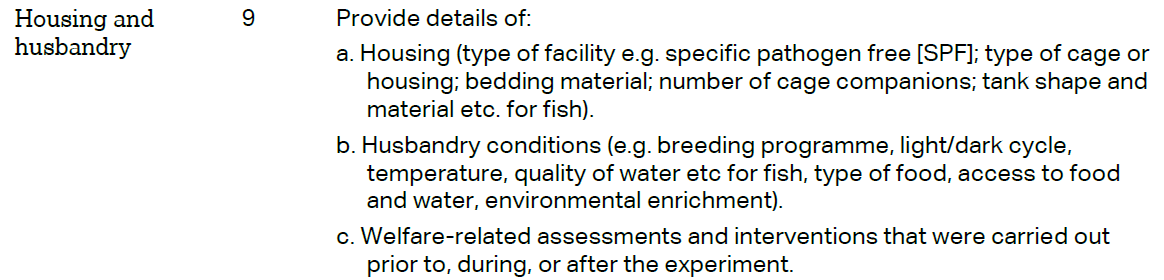 | Paragraph 2  Paragraph 2  Paragraph 12 | |
| --- | --- | --- |
| 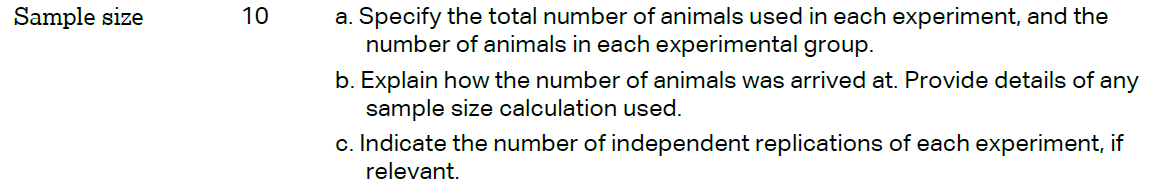 | Paragraph 12 | |
| 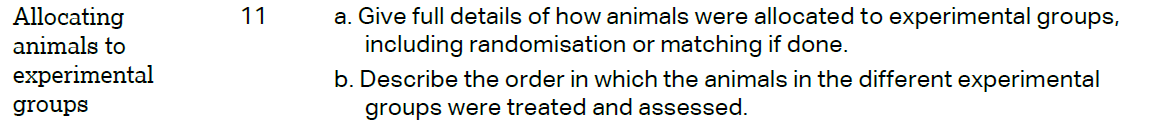 | Paragraph 12 | |
| 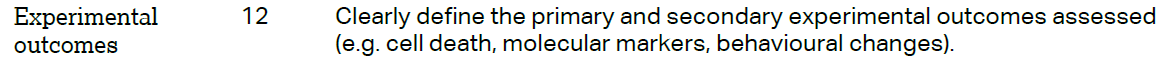 | Paragraph 13 | |
| 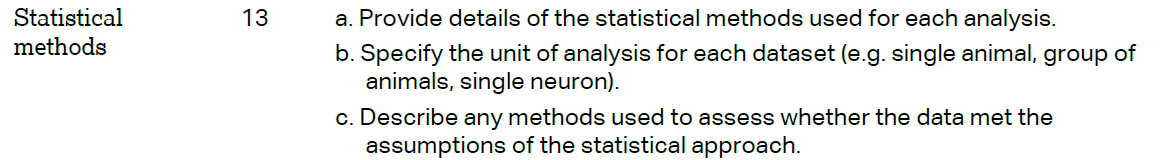 | Paragraph 14 | |
| RESULTS |  | |
| 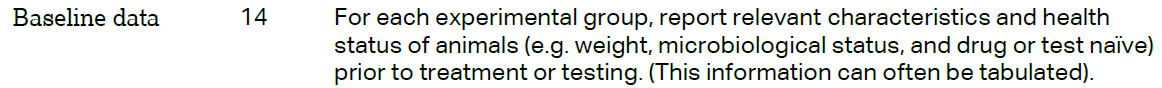 |  | |
| 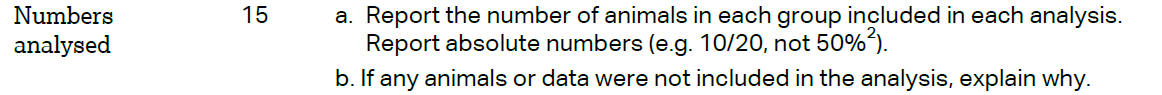 | Paragraph 8 | |
| 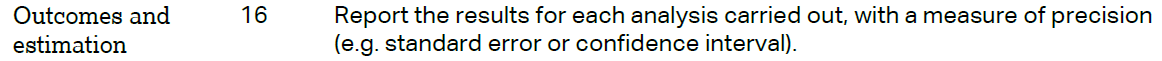 | Paragraph 8 | |
| 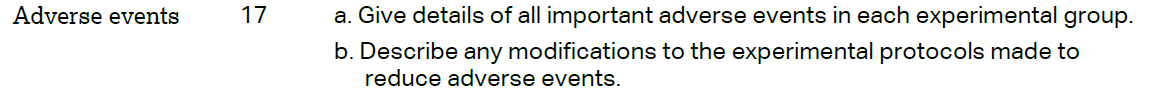 |  | |
| DISCUSSION |  | |
| 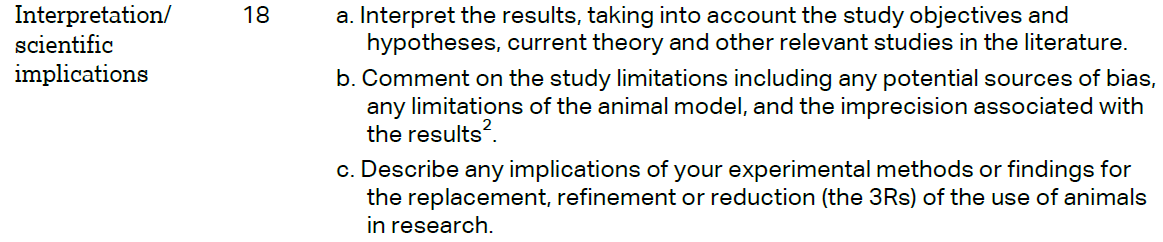 | Paragraph 2 | |
| 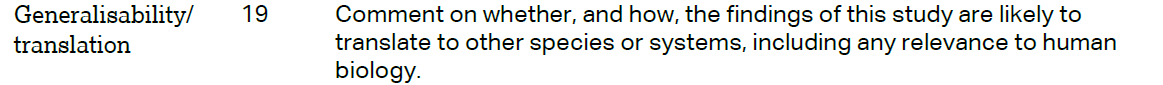 |  | |
| 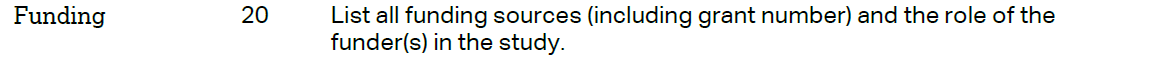 | |  |


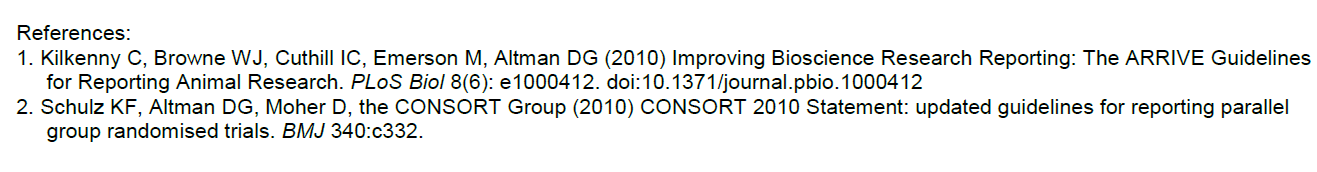

Supplement: S1 Checklist — (DOCX) [file pone.0207760.s001.docx]
